# Cervical cancer screening among female health workers: Evidence from a secondary health facility in Northern Malawi

**DOI:** 10.1016/j.pmedr.2023.102581

**Published:** 2023-12-27

**Authors:** Atusaye Mwalwanda, Elton Chavura, Master R.O. Chisale, Balwani Chingatichifwe Mbakaya

**Affiliations:** a University of Livingstonia, Department of Public Health, Malawi; b Mzuzu University, Faculty of Environmental Science, Mzuzu, Malawi; c Mzuzu University, Faculty of Science Technology and Innovations, Biological Sciences, Mzuzu, Malawi; d Luke International Malawi, Malawi

**Keywords:** Cervical cancer, Screening, Female health workers, Knowledge, Attitude, Practices

## Abstract

Cervical cancer is preventable and curable if identified early. Although health workers have a critical role in influencing beliefs and practices of the entire population, some studies have shown paradoxical efforts among female health workers themselves-a threat towards attainment of the triple-intervention strategy as propagated by the World Health Organization (WHO). The study aimed to assess knowledge, attitudes and practices of cervical cancer screening among female health workers.

The study used a descriptive cross-sectional design. Data entry and analysis were carried out using IBM Statistical Package for Social Scientists (SPSS) version 20.0 (SPSS, Michigan Avenue, Chicago, IL, USA).

The response rate was 65.4 % and mean age of 32 years with standard deviation=±8.397. The majority of participants were nurses n = 31 (43.3). There was poor knowledge on signs/symptoms and risk factors as only 15.7 % were aware of multiple sexual partners, 7.1 % for each early sexual encounter and uncircumcised men as risk factors. Regarding willingness, 77.1 % were willing to have the screening test. Their practices were rated poor as only 35.7 % (n = 25) had ever undergone screening within the past 6 years.

There was poor knowledge, fair attitudes and poor practices of cervical cancer screening among female health workers. As part of the efforts to achieve sustainable development goal (SDG) target 3.4 and the attainment of WHO 90–70-90 target by 2030; this study recommends scaling up health education, social mobilization and Human Papillomavirus (HPV) vaccinations to support awareness, detection and treatment of cervical cancer. Acceptability of the current screening methods must be further explored.

## 1 Background

Cervical cancer is preventable and in most cases curable if identified in its early stage (Getahun, 2013). Yet it remains one of the most common cancers and causes of related death in women across the globe (Arbyn, 2020). It has been observed through studies that cervical cancer screening, even when done once, reduces the burden of cervical cancer mortality (Atashili, 2011). Over the past years, the incidence of cervical cancer has decreased in developed countries mainly due to increased awareness and more effective screening and prevention strategies employed in these areas together with vaccine contribution (Torre, 2017). Cervical cancer is one of the most common cancers worldwide among women and ranks as the fourth most frequent cancer among women in the world (Arbyn, 2020). According to Information Commissioner‘s Office /Integrated Agency for Research on cancer (ICO/IARC), (2019) (Bray et al., 2018) the world has a population of 2,784 million women aged 15 and above at risk for developing cervical cancer and estimates that every year 569,847 women are diagnosed with cervical cancer and 311, 365 die from the disease. The WHO, (2020) (Gültekin, 2020) global strategy to eliminate cervical cancer aims to accomplish the 90–70-90 target by 2030. The first 90 % is for girls to be fully vaccinated with HPV vaccine by age of 15 years, and 70 % of women are to be screened by the age of 35 years and again by 45 years the last 90 % of women identified with cervical disease receive treatment.

Different studies have shown that very few women in sub-Sahara Africa and other developing countries are screened for cervical cancer and low levels of awareness and poor knowledge of cervical cancer together with unavailability and inaccessibility of cervical cancer screening services are responsible for the small number of women being screened (Abotchie and Shokar, 2009). It has also been estimated that from 2013, cervical cancer incidence and mortality will continue to rise in sub-Saharan Africa over a period of 20 years (De Vuyst, 2013). In Malawi cervical cancer mortality rate is estimated at about 49.8 per 100,000 populations and that over 4.76 million women aged between 15 years and older are at risk of developing cervical cancer in Malawi (Malawi National Statistics Office (NSO), 2017). This is in spite of the national *cervical cancer screen and treat programme* using visual inspection with acetic acid (VIA) currently being implemented in Malawi (Msyamboza, 2017). Women no younger than 30 years are offered three free smears, with a 10 years interval in between each smear (Ferlay et al., 2012) and those screened for the first time at the age of 55 or more have only one smear if the first smear is normal (Msyamboza, 2016). In 2013, with the support from the Global Alliance for vaccines and immunization, Malawi introduced the HPV vaccination demonstration project in Rumphi and Zomba among girls aged 9–13 years and so far the project has covered over 20 districts. Malawi reported 4,163 new cervical cancer cases in 2018 representing 72.9 of Age-standardized incidence rate, with crude incidence rate of 43.1 ranking cervical cancer as the first leading cause of female cancer death in Malawi. The 2018 estimates also reviewed that about 2,800 cervical cancer deaths occur in Malawi representing 54.5 of Age-standardized mortality rate, with crude mortality rate of 54.5 (Bruni et al., 2019).

Considerable differences persist across countries’ screening guidelines, even among comparable systems. In Malawi, the cervical cancer screening and preventive treatment program is informed by a robust screening data infrastructure and the following two instruments namely: The Malawi cervical cancer operational guidelines, and the National Cervical Cancer Control Strategy 2016––2020 (Gerstl, 2022). The strategy incorporates cervical cancer prevention and control through the promotion of HPV vaccine and integration of cervical cancer screening into Human Immunodeficiency virus (HIV) care. The current recommendations for screening in Malawi are that women aged between 25 and 49 years are screened once every 3–––5 years. Owing to high mortality related to cervical cancer (Ferlay, 2018), an increased susceptibility to HPV infection among HIV infected women (Dreyer, 2018) and high prevalence of Human Immunodeficiency Virus (HIV) infection amongst women of child-bearing age (Malawi National Statistics Office (NSO), 2017); (UNAIDS, UNAIDS Malawi Overview, 2018), the guidelines further recommended routine screening among HIV positive women once every 12 months.

In Malawi, studies have shown that cervical cancer screening uptake among health workers is associated with perceived barriers and susceptibility, where fear to be screened by fellow staff and health workers feeling safe that they cannot be affected with the cervical cancer respectively. All of these lead health workers not to engage in cervical cancer screening practices (Parmar and Taylor, 2010). It is for this reason that this study targeted health workers that have a critical role in influencing beliefs and practices of the entire population. Some studies have shown paradoxical efforts among female health workers themselves-a threat towards attainment of the triple-intervention strategy as propagated by the World Health Organization (WHO). When diagnosed early with cervical cancer is one of the most successful preventable forms of cancer with early detection through screening. However, some previous study findings (Chadza et al., 2012) have reported that Malawian women delay seeking medical attention due to limited knowledge on signs and symptoms hence the late diagnosis.

## 2 Methodology

### 2.1 Study design

This study used a descriptive cross-sectional design.

### 2.2 Study setting

The study was conducted at Karonga District Hospital. Karonga District (KDH) is in the northern part of Malawi, bordered by Lake Malawi to the East, by Songwe River (border with Tanzania) to the North and Nyika plateau and highlands to the west and south.

### 2.3 Study populations

The district hospital offers cervical cancer screening services to everyone who is eligible for free. Respondents to the study included female health workers such as nurses, clinicians, hospital attendants, laboratory technicians, health surveillance assistants (H.S.A) and other support staff.

### 2.4 Sample size

A sample size of 70 female health workers was involved in this study, as determined by Cochran formula. According to the office of human resource and 2021 facility registry, there were 302 health workers at Karonga District Hospital, where 155 were males and 147 females across different professional cadres. Cochran‘s formula was laid out as follows:

$$n=\frac{n_{0}}{1+\frac{\left(n_{0}-1\right)}{N}}$$

Where n_0_ was given by:

$$n_{0}=\frac{z^{2}pq}{e^{2}}\;=\;n_{o}=\frac{1.96^{2}\left(0.5\right)\left(0.5\right)}{0.05^{2}}\;=\;384$$

$$n=\frac{n_{0}}{1+\frac{\left(n_{0}-1\right)}{N}}=n=\frac{384}{1+\frac{\left(384-1\right)}{147}}\;=\;n=107$$

However, out of the 107 sample size only 70 female health participants participated in this study from different professionals.

### 2.5 Sampling technique

The study used simple random sampling where all female health workers at KDH were chosen from the study and each participant had equal chances of being selected until the required sample was reached. Each female health worker was assigned a number and was placed in a container and then picked any number at random. The female health workers corresponding to the numbers picked were included in the sample.

### 2.6 Inclusion and exclusion criteria

All female health workers working at KDH qualified for inclusion into this study. However, female health workers who declined consent for participation and those who were not on duty on the days of data collection such as being off duty and on leave, were excluded from the study.

### 2.7 Validity and reliability of measures

The questionnaire was initially developed in English and subsequently translated into local language. Different relevant literature was reviewed to develop a tool that addresses the objectives of the study. Knowledge about cervical cancer was measured by using knowledge questions about risk factors, signs and symptoms of cervical cancer. The questionnaire was pre-tested at a different district hospital.

### 2.8 Ethical considerations

The purpose of the study was explained to Karonga District Hospital authorities for the permission to conduct the study at their facility. The participants were comprehensively informed on the purpose of the study. Participants were assured of confidentiality on the information obtained. All participants signed a written informed consent. Ethical approval was granted by the University of Livingstonia, faculty of Applied Sciences, Department of Public Health, with approval number 0421.

### 2.9 Data collection

The questionnaire was used in this research which was administered to individuals in person. The questionnaire consisted of structured (closed ended) questions where respondents were required to select answers from the given choices. The questionnaire took approximately 15 min to administer it.

### 2.10 Data analysis

Data collected for analysis were fed into computers and processed through SPSS version 20 for easy analysis and interpretation of results. Descriptive analysis was performed to generate frequencies, percentages, mean and standard deviations. Research findings are presented through charts, tables and percentages.

## 3 Results

### 3.1 Main findings

#### 3.1.1 Demographic data

A total of 70 out of 107 female health workers participated in the study with a response rate of 65.4 %. Participant‘s age ranged from 19 to 59 years with mean age of 32 years and standard deviations=±8.397. Of the respondents, 32(45.7 %) were married followed by single 26 (37.1 %). Majority of participants were Nurses 31 (44.3 %) followed by Hospital Attendants 17 (24.3 %) and HSA 11(15.7 %), *refer to* Table 1.

**Table 1:** Demographic data of female health workers at Karonga District Hospital- February 2021.

**Variable**	**Mean**	**(±SD)**
**Age**	32	± 8.397
**Variable**	**Frequency (n)**	**Percentage (%)**
**Marital status of respondents**		
Married	32	45.7
Single	26	37.1
Divorced	5	7.1
Widow	7	10.0
**Qualification of respondents**		
Malawi School Certificate of Education	29	41.4
Certificate	9	12.9
Diploma	23	32.9
Bachelor's degree	9	12.9
**Profession of respondents**		
Nurse and Midwife	31	44.3
Laboratory Technician	2	2.9
Pharmacy technician	3	4.3
Medical assistant	2	2.9
H.S.A	11	15.7
Hospital attendant	17	24.3
Counsellor	1	1.4
Data Clerk	3	4.3

#### 3.1.2 Knowledge (signs/symptoms, risk factors) and source of information about cervical cancer

All of the respondents, 70 (100 %) heard about cervical cancer. About 53 (75.7 %) respondents knew that HPV causes cervical cancers. The main source of information on cervical cancer and screening was obtained at the health facility 39 (55.7 %). However, the majority of respondents rarely had cervical cancer education represented with 59 (84.3 %), *refer to* Table 2*.* Majority of participants 53 (80.3 %) had good knowledge on bleeding between periods, besides poor knowledge on bleeding after intercourse and lower abdominal pains as signs and symptoms of cervical cancer represented with 40 (60.6 %) and 44(66.7 %) respectively. Additionally, most participants 42(60 %) were unaware that foul discharge is a sign of cervical cancer, *refer to* Table 3*.* Majority of participants identified unprotected sexual intercourse 36 (51.4 %) as a risk factor of cervical cancer, *refer to* Table 2*.*

**Table 2:** General knowledge on cervical cancer among female health workers at Karonga District Hospital- February 2021.

**Variable**	**Frequency (n)**	**Percentage (%)**
**Ever heard of cervical cancer and screening**		
Yes	70	100.0
**Source of information about cervical cancer**		
Parents	1	1.4
school (primary and secondary)	3	4.3
College and university	15	21.4
Radio	12	17.1
health facility	39	55.7
**How often do you have cervical cancer education**		
Once a year	4	5.7
once a month	1	1.4
two times a month	4	5.7
two times in six months	2	2.9
Rarely	59	84.3
**Can human papillomavirus cause cervical cancer**		
True	53	75.7
False	2	2.9
do not know	15	21.4
**Risk factors of cervical cancer**		
Unprotected sex	36	51.4
Multiple partners	11	15.7
Early sexual debut	5	7.1
uncircumcised men	5	7.1
Do not know	13	18.6
**Is cancer of cervix preventable**		
Yes	68	97.1
No	2	2.9

**Table 3:** Knowledge on signs and symptoms of cervical cancers among female health workers at Karonga District Hospital-February 2021.

					**Profession**					**Total**
		**Nurse**	**Laboratory Tech**	**Pharmacy tech**	**Medical assistant**	**HSA**	**Hospital attendant**	**Counsellor**	**Data Clerk**	
**Signs and symptoms of cervical cancer**										
Bleeding in between period	Count	27	1	1	2	8	13	1	0	53
	% Within Signs/Symptoms	50.9	1.9	1.9	3.8	15.1	24.5	1.9	0	
	% Within Profession	87.1	50	100	100	88.9	76.5	100	0	
	% Of Total	40.9	1.5	1.5	3	12.1	19.7	1.5	0	80.3
Foul discharge	Count	21	0	0	0	3	4	0	0	28
	% Within Signs/Symptoms	75	0	0	0	10.7	14.3	0	0	
	% Within Profession	67.7	0	0	0	33.3	23.5	0	0	
	% Of Total	31.8	0	0	0	4.5	6.1	0	0	42.4
Bleeding after intercourse in women	Count	26	2	0	2	2	8	0	0	40
	% Within Signs/Symptoms	65	5	0	5	5	20	0	0	
	% Within Profession	83.9	100	0	100	22.2	47.1	0	0	
	% Of Total	39.4	3	0	3	3	12.1	0	0	60.6
Lower abdominal pains	Count	21	1	1	1	4	12	1	3	44
	% Within Signs/Symptoms	47.7	2.3	2.3	2.3	9.1	27.3	2.3	6.8	
	% Within Profession	67.7	50	100	50	44.4	70.6	100	100	
	% Of Total	31.8	1.5	1.5	1.5	6.1	18.2	1.5	4.5	66.7

#### 3.1.3 Attitude towards cervical cancer

Regarding willingness, majority of the participants 54 (77.1 %) were willing to get the VIA test. Out of the 16 participants that had no intention of getting the test identified that had no signs and symptoms of cervical cancer 7 (43.8 %) and painful procedure 4 (25 %) as common reasons, *refer to* Table 4*.*

**Table 4:** Attitude towards cervical cancer among female health workers at Karonga District Hospital-- February 2021.

**Variable**	**Frequency (n)**	**Percentage (%)**
**Are you willing to get Visual inspection with an acetic acid test?**		
Yes	54	77.1
No	16	22.9
**If no what are the reasons for not willing?**		
painful instrument	4	25.0
No signs of cervical cancer	7	43.8
fear of procedure	1	6.3
procedure not safe	1	6.3
fear from friends	1	6.3
lack of privacy	1	6.3
**What do you think of VIA provider’s attitudes at your facility?**		
lack of privacy	24	38.7
Friendly	38	61.3
Do not know	8	10.0
**What do you think of the cervical cancer screening procedure?**		
Safe	38	54.3
Not safe	29	41.4
Do not know	3	4.3
**Does feeling healthy make you free from cervical cancer?**		
Yes	25	35.7
No	45	64.3
**How embarrassed are you to get a visual inspection test?**		
not embarrassed	36	51.4
Very embarrassed	34	48.6
**If very embarrassed, what are the reasons?**		
Procedure done by male nurses/doctors	12	35.3
Procedure done by fellow female nurses/doctors	14	41.2
Positive results	8	23.5

#### 3.1.4 Practice towards cervical cancer and availability of cervical cancer services

Although 57 (81.4 %) participants agreed to have cervical cancer services at their facilities, only 25 (35.7 %) of the participants had any VIA test in the past 6 years despite a slight increase in the numbers of health workers attending the test within each 3 years from 2009 to 2021. Out of the 70 participants, 13 (18.6 %) did not have the VIA test facility at their site. Out of 45 (64.3 %) participants who did not have the test in the past 6 years, gave the following reasons; majority 13 (28.9 %) had fear of outcome, followed by participants that had no symptoms 11 (24.4 %), *refer to* Table 5.

**Table 5:** Practice towards cervical cancer among female health workers at Karonga District Hospital- February 2021.

**Variable**	**Frequency (n)**	**Percentage (%)**
**Do you have a cervical cancer test at your facility?**		
Yes	57	81.4
No	13	18.6
**When did you have your last visual inspection test done?**		
never done	31	44.3
2019 to 2021	13	18.6
2016 to 2018	12	17
2013 to 2015	11	15.7
2009 to 2011	3	4
**Have you had any visual inspection test in the last 6 years?**		
Yes	25	35.7
No	45	64.3
**If no why?**		
Not convenient /no time	5	11.1
Fear of procedure	8	17.8
Fear of outcome	13	28.9
No symptoms hence no need of the test	11	24.4
Lack of resources	8	17.8
**Where do you prefer to have the VIA?**		
Private clinic	8	11.4
District hospital	37	52.9
Self-sampled vaginal swab	13	18.6
Health centre	12	17.1

Regarding the profession, those that had any VIA test in the last 6 years, out of the 8 professionals only Nurses, Hospital Attendants and HSA had VIA test done. Additionally, Out of the 57 participants that agreed to have cervical cancer services at their facilities, only 23 participants had a VIA test within the last 6 years. As regards to willingness and practice, out of the 54 participants willing to have the test only 24 participants had the test within the last 6 years, *refer to* Table 6.

**Table 6:** VIA Test*Cervical Cancer Screening test facility*Willingness among female health workers at Karonga District Hospital- February 2021.

Profession of respondents * Have you had any visual inspection test in the last 6 years.				
	**Have you had any visual inspection tests in the last 6 years?**			**Total**
Profession of respondents	YES	NO		
Nurse and Midwife	14	17		31
Laboratory Technician	0	2		2
Pharmacy technician	0	3		3
Medical assistant	0	2		2
HSA	2	9		11
Hospital attendant	9	8		17
Counsellor	0	1		1
Data Clerk	0	3		3
**Total**	25	45		70
Do you have a cervical cancer test at your facility * Have you had any VIA test in the last 6 years?				
		**Have you had any VIA test in the last 6 years?**		**Total**
		YES	NO	
**Do you have a cervical cancer test at your facility**	Yes	23	34	57
	No	2	11	13
**Total**		25	45	70
Are you willing to get VIA with acetic acid tests * Have you had any VIA tests in the last 6 years?				
		**Have you had any VIA tests in the last 6 years?**		**Total**
		YES	NO	
**Are you willing to get Visual inspection with an acetic acid test**	Yes	24	30	54
	No	1	15	16
**Total**		25	45	70

## 4 Discussion

In this study a total of 70 female healthcare workers participated. The results in this study revealed that most of the female health workers ever heard of cervical cancer (100 %). About 97.1 % correctly identified cervical cancer as preventable and 75.7 % noted HPV as the cause. Similar study done in Ethiopia, Addis Ababa among women showed a lower percentage (42.7 %) of those who had ever heard of cervical cancer (Getachew, 2019). The difference could be as a result of the Ethiopian study which was conducted on community women rather than the current study where participants were health workers that are expected to have been much exposed to the information.

This study found that the main source of information was health facilities (55.7 %). Despite health facilities being the main source, this study revealed that 84.3 % of respondents rarely had cervical cancer and screening education as part of the facility program. The current results are similar to the study conducted at Ibadan in Nigeria that revealed cervical cancer was never a topic of discussion at the clinic (Ndikom and Ofi, 2012). Therefore, this study learnt that health workers at KDH lack in-depth understanding of information as they are not frequently updated and this might have been one of contributing factors to inadequate knowledge on risk factors, signs/symptoms and low uptake of screening. This is also alluded to by a study conducted in northern Malawi (Kaseka, 2022) that emphasized the need to have a well organised cervical cancer screening and Pap smear test and programs in Malawi in order to reduce the prevalence of cervical cancer.

Regarding cervical cancer risk factors, this study revealed low knowledge as only 51.4 %, 18.6 %, 15.7 % and 7.1 % identified unprotected sex, multiple partners, early sexual debut and uncircumcised men respectively as risk factor. These results are different from a study conducted in Northern Uganda, in which 99 % identified having multiple sexual partners as risk factors (Obol, 2021). This study revealed that most participants were knowledgeable on unprotected sex as a main risk factors. This study also revealed that most of the participants had little information about foul discharge (42.4 %), bleeding after intercourse (60.6 %) and Lower abdominal pains (66.7 %) as signs and symptoms of cervical cancer. The finding of this study supports a previous study finding (Chadza et al., 2012) which reported that Malawian women delay seeking medical attention due to limited knowledge on signs and symptoms hence the late diagnosis. While the majority had more knowledge of bleeding in between periods (80.3 %) as the main sign and symptom. The lesson learnt from this study implies that health workers at KDH would suspect cervical cancer more if bleeding between periods is observed, hence contributing to factors that affect low uptake of screening. Therefore, providing health education about cervical cancer prevention to health workers such as hospital facilities might increase their knowledge and awareness of the disease, subsequently increasing the accessibility and practice. This is supported in a study conducted in northern Malawi on Histopathalogical profile of cervical biopsies that also revealed the need to create community awareness and strengthen early cervical cancer screening for Malawi to have better outcomes (Kaseka, 2022).

Health care attitude and willingness towards cervical cancer would reflect a positive attitude to increasing cervical cancer screening. This study revealed that 77.1 % were willing to get cervical cancer screening. The results were different from a study conducted in India where 32 % of women were willing to undergo screening (Chandrika et al., 2020) the reason being that previous study involved community women while current study involved female health workers considered to have much information. Regarding those that were not willing (22.9 %), this study revealed that no signs and painful instrument/procedure as contributing factors. The lesson learnt revealed that willingness was not a contributing factor to practice as only 44.4 % of the 54 participants that were willing had done the test in the past 6 years.

This study also revealed poor practice among female health workers towards cervical cancer as only 35.7 % of participants were screened in the past 6 years, despite all participants ever heard of cervical cancer and screening. This study also revealed a slight increase in change of numbers of women testing within each 3 years from 2009 to 2021. This is similar to a study conducted in Mangochi (Malawi) among women of child bearing age that had even lower results than the current study with only 13.2 % had ever been screened (Mpachika, 2021). Profession was a contributing factor to cervical cancer screening as more Nurses and Hospital Attendants indicated to have gone for VIA test for the past 6 years. This might be due to the nature of nursing courses while Hospital Attendants support nurses in management of cervical cancer clients in the ward.

This study also learnt that availability of the cervical cancer test facility was not a contributing factor to practice as only 40.4 % had the test in the past 6 years for those who had services at their facility. Therefore, poor practice observed in this study is not entirely dependent on availability of services, attitude and profession but also influenced by broader factors such as knowledge in terms of risk factors, signs and symptoms of cervical cancer. This study revealed lack of knowledge, fear of outcome, painful procedure and no signs/symptoms as contributing factors for low uptake of screening at KDH. Lastly, this study also revealed that marital status was a contributing factor to cervical cancer screening with 72 % of participants that had done the test were married. Hence, there is also a need to target more unmarried female health workers for cervical cancer services at KDH and surrounding health centres.

## 5 Limitations

The study obtained data from one health facility only, and that may limit generalisation of results. Due to Covid-19 restrictions, there was some resistance for the facility and respondents to take part in research. Lack of previous research studies on cervical cancer screening among female health workers was among the main limitations that has contributed to scarcity of information. Finally, a small sample size could limit generalisation

## 6 Conclusion and recommendations

This study demonstrates that there is poor knowledge, fair attitudes and poor practices of cervical cancer screening among female health workers. As part of the efforts to achieve sustainable development goal (SDG) target 3.4 which aims to reduce preventable deaths by one third by 2030 and also to achieve the WHO 90–70-90 target by 2030; there is need to strengthen awareness to improve knowledge, attitude and practice on cervical cancer screening. This study recommends scaling up health education and social mobilization and HPV vaccinations to support awareness, detection and treatment of cervical cancer. Acceptability of the current screening methods must be further explored.

## Author contribution

All authors equally contributed to this manuscript. Their various expertise greatly contributed toward the quality of this manuscript.

All authors read and approved the submission of this manuscript.

## Funding

There was no any funding towards this study.

## Competing interest

All authors declare no competing interest.

## CRediT authorship contribution statement

**Atusaye Mwalwanda:** Data curation, Formal analysis, Methodology, Writing – original draft. **Elton Chavura:** Data curation, Formal analysis, Methodology, Supervision, Validation, Writing – review & editing. **Master R.O. Chisale:** Data curation, Formal analysis, Investigation, Methodology, Visualization, Writing – review & editing. **Balwani Chingatichifwe Mbakaya:** .

## Declaration of competing interest

The authors declare that they have no known competing financial interests or personal relationships that could have appeared to influence the work reported in this paper.

## Data Availability

Data will be made available on request.
